# Clinical significance of APOB inactivation in hepatocellular carcinoma

**DOI:** 10.1038/s12276-018-0174-2

**Published:** 2018-11-14

**Authors:** Gena Lee, Yun Seong Jeong, Do Won Kim, Min Jun Kwak, Jiwon Koh, Eun Wook Joo, Ju-Seog Lee, Susie Kah, Yeong-Eun Sim, Sun Young Yim

**Affiliations:** 1 Department of Systems Biology, The University of Texas MD Anderson Cancer Center, Houston, TX USA; 2 Department of Pathology, Seoul National University College of Medicine, Seoul, Korea; 3 Department of Gynecology, School of Medicine, Kyung Hee University, Seoul, Korea; 4 Department of Internal Medicine, School of Medicine, Kyung Hee University, Seoul, Korea; 5 Department of Internal Medicine, Korea University, College of Medicine, Seoul, Korea

## Abstract

Recent findings from The Cancer Genome Atlas project have provided a comprehensive map of genomic alterations that occur in hepatocellular carcinoma (HCC), including unexpected mutations in apolipoprotein B (*APOB*). We aimed to determine the clinical significance of this non-oncogenetic mutation in HCC. An Apob gene signature was derived from genes that differed between control mice and mice treated with siRNA specific for Apob (1.5-fold difference; *P* < 0.005). Human gene expression data were collected from four independent HCC cohorts (*n* = 941). A prediction model was constructed using Bayesian compound covariate prediction, and the robustness of the APOB gene signature was validated in HCC cohorts. The correlation of the APOB signature with previously validated gene signatures was performed, and network analysis was conducted using ingenuity pathway analysis. APOB inactivation was associated with poor prognosis when the APOB gene signature was applied in all human HCC cohorts. Poor prognosis with APOB inactivation was consistently observed through cross-validation with previously reported gene signatures (NCIP A, HS, high-recurrence SNUR, and high RS subtypes). Knowledge-based gene network analysis using genes that differed between low-APOB and high-APOB groups in all four cohorts revealed that low-APOB activity was associated with upregulation of oncogenic and metastatic regulators, such as *HGF*, *MTIF*, *ERBB2*, *FOXM1*, and CD44, and inhibition of tumor suppressors, such as *TP53* and *PTEN*. In conclusion, APOB inactivation is associated with poor outcome in patients with HCC, and APOB may play a role in regulating multiple genes involved in HCC development.

## Cancer: Spotlighting hidden cancer-causing genes

Mutation of a gene with no clear role in tumor development triggers a cascade of reactions that can cause liver cancer. Recent genome-wide analyses searching for genes connected to development of hepatocellular carcinoma, the most common type of liver cancer, have turned up some unexpected genes, such as the fat metabolism gene apolipoprotein A (*APOB*). To discover how *APOB* is related to liver tumor development, Sun Young Yim at the University of Texas MD Anderson Cancer Center, Houston, USA, and coworkers compared whole-genome profiles from human cancer patients with those of mice, in which cancer-related genetic patterns are better characterized. They found that mutation of *APOB* was associated with switching on of cancer-promoting genes, and switching off of genes that suppress tumor growth. These results reveal a behind-the-scenes regulator of cancer development.

## Introduction

Liver cancer is the seventh most common cancer globally^1,2^, and hepatocellular carcinoma (HCC) represents 75% of cases of primary liver cancer^3^. The incidence of liver cancer has increased in the United States over the past 25 years (40,710 estimated new cases in 2017)^4^. Incidence and mortality rates for liver cancer are expected to double over the next 10–20 years^5,6^. HCC is the second most lethal cancer type, and the 5-year overall survival rates in the United States are only approximately 11%^7^. Although sorafenib and regorafenib are approved molecular targeted therapies for HCC, they have shown only limited clinical benefit^8,9^. Lack of molecular classification and clinical heterogeneity in HCC are likely the reasons for underdevelopment of standardized treatment for this malignant disease.

Recent advances in sequencing technologies have allowed for improved analysis of multiple cancer genomes. To gain a comprehensive view of the genetic alterations that occur during the HCC development, many researchers have analyzed the HCC genome using whole-exome or whole-genome sequencing strategies^10–16^. These studies have reported frequently mutated genes, such as *TERT*, *TP53*, *CTNNB1*, *ARID1A*, *ARID2*, *NFE2L2*, and *KEAP1*. Not surprisingly, many of these mutated genes are known tumor suppressors or tumor-driving oncogenes. Recent results from The Cancer Genome Atlas (TCGA) project also identified a similar spectrum of mutated genes in HCC^17^. However, in addition to identifying mutations in oncogenes and tumor suppressor genes, TCGA identified frequent mutations in albumin (*ALB*) and apolipoprotein B (*APOB*), which are not frequently mutated in other cancer types, suggesting that they may play unique roles in the development of HCC. Interestingly, pathogenic mutations in APOB were previously recognized in patients with familial hypobetalipoproteinemia (FHBL), which is characterized by reduced plasma levels of total cholesterol and low-density lipoprotein cholesterol^18^. Individuals with FHBL attributed to mutations in APOB are prone to hepatic steatosis, liver cirrhosis, and hepatocarcinoma^19^. Thus, frequent mutation of these serum proteins in HCC may not be accidental. However, the clinical relevance of loss of these serum proteins is currently unknown.

In previous studies^20–22^, we demonstrated the feasibility of “comparative systems genomics,” which involves cross-species comparison of gene expression data from mouse models and human patients with HCC to stratify patients into molecularly defined subtypes according to well-characterized gene expression signatures from the mouse models. In the present study, we expanded this approach to address the clinical relevance of loss of APOB in HCC in multiple cohorts. Furthermore, we uncovered a potential connection between APOB and regulation of oncogenic signaling pathways.

## Materials and methods

### Gene expression data from mouse livers

Generation of homozygous *Ldlr*^–/–^ *CETP*^*+/+*^ mice and gene expression data from mouse livers were described in an earlier study^23^. Briefly, total RNA was extracted from liver tissues of *Ldlr*^–/–^ *CETP*^*+/+*^ and wild-type mice after silencing of Apob expression by intravenous injection of two independent mouse Apob-specific siRNAs^23^. Gene expression data were generated using Merck/Affymetrix mouse 1.0 custom arrays with >25,000 genes. Data are available in the National Center for Biotechnology Information Gene Expression Omnibus (GEO) database (GSE23088).

### Gene expression data from human HCC tissues and clinical data

Gene expression data from The University of Texas MD Anderson Cancer Center (MDACC) cohort were described in an earlier study^24^. Briefly, tumor specimens and clinical data were obtained from patients with HCC who had undergone hepatectomy. Eighty-eight surgically removed frozen HCC specimens were used for the microarray experiments. Samples were frozen in liquid nitrogen and stored at –80 °C until RNA extraction. The study protocols were approved by the institutional review board, and all participants provided written informed consent. Gene expression data were generated using the Illumina microarray platform human-6 v.4. Patients were monitored prospectively at least once every 3 months after surgery. The primary microarray data are available in the National Center for Biotechnology Information GEO public database (accession number GSE43619).

Gene expression data from the Samsung and Fudan cohorts were also described in previous studies (GEO accession numbers GSE36376 and GSE14520)^25–27^. All study protocols for these cohorts were approved by the institutional review boards. In addition, we included gene expression data from the HCC project of TCGA^17^ in our analysis. Table 1 shows the pathologic and clinical characteristics of the patients in all four cohorts. All patients had undergone surgical resection as their primary treatment.

**Table 1:** Baseline clinical and pathologic features of patients with hepatocellular carcinoma in all four cohorts

Variable	Cohort, *n* (%)			
	MDACC	Samsung	Fudan	TCGA
Total number of patients	88	240	242	371
Sex^a^				
Male	73 (83)	199 (83)	211 (87)	73 (20)
Female	15 (17)	41 (17)	31 (13)	15 (4)
NA				283 (76)
Age (years)				
Median	58	53	50	61
Range	29–77	17–76	21–77	17–90
AFP				
>300 ng/ml	23 (26)	78 (33)	110 (45)	
≤300 ng/ml	64 (73)	154 (64)	128 (53)	
NA	1 (1)	8 (3)	4 (2)	371 (100)
HBV				
+	80 (91)	206 (86)	242 (100)	
–	8 (9)	34 (14)	0 (0)	
NA				371 (100)
AJCC stage				
I	68 (77)	102 (43)	96 (40)	164 (44)
II	13 (15)	99 (41)	78 (32)	82 (22)
III	7 (8)	34 (14)	51 (21)	78 (21)
IV	0	5 (2)	0 (0)	5 (1)
NA			17 (7)	42 (11)
BCLC stage				
0	4 (5)	1 (1)	20 (8)	
A	53 (60)	138 (57)	152 (63)	
B	26 (30)	91 (38)	24 (10)	
C	5 (6)	10 (4)	29 (12)	
D			0 (0)	371 (100)
NA			17 (7)	
Death	17 (19)	108 (45)	96 (40)	121 (33)
Median follow-up duration	31.5 months	91 months	51.6 months	19.7 months

*MDACC* The University of Texas MD Anderson Cancer Center, *TCGA* The Cancer Genome Atlas, *NA* not applicable, *AFP* alpha-fetoprotein, *HBV* hepatitis B virus, *AJCC* American Joint Committee on Cancer staging system, *BCLC* Barcelona Clinic Liver Cancer staging system

^a^Patient sex was not available for patients who underwent liver transplantation

### Clinically defined HCC molecular subtypes and associated gene expression signatures

The National Cancer Institute proliferation (NCIP)^20,28,29^, hepatic stem cell (HS)^21^, Seoul National University recurrence (SNUR)^30^, and recurrence-risk score (RS)^31^ HCC subtypes and associated gene signatures were described in earlier studies.

#### Cell lines and cell cultures

JHH4 and JHH6 cells were obtained from the Health Science Research Resources Bank (Osaka, Japan), and HepG2 cells were obtained from the American Type Culture Collection (Manassas, VA). JHH4 cells were cultured in Eagle’s minimal essential medium containing heat-inactivated 10% fetal bovine serum (FBS) and 1% antibiotics (Invitrogen, Carlsbad, CA, USA). JHH6 cells were cultured in Williams’ medium E containing 10% FBS and 1% antibiotics. HepG2 cells were cultured in Dulbecco’s modified Eagle’s medium with high glucose containing 10% FBS and 1% antibiotics (Invitrogen).

#### Silencing APOB expression

siAPOB and shAPOB were purchased from Sigma. For siRNA-based silencing of APOB expression, JHH4 and JHH6 cells were transfected with the indicated siRNA using Mission siRNA transfection reagent (Sigma, St. Louis, MO). For lentiviral shRNA-based silencing of APOB, shAPOB-1, shAPOB-2, and shAPOB-3 were used. Sigma Mission non-targeting shRNA control was used as the control. The siRNA and shRNA sequences are available in Supplementary Table 1. 293 T cells were co-transfected with APOB or control shRNA plasmids along with packaging plasmids (psPAX2) and envelope plasmid (pMD2.G) using X-tremeGENE HP DNA transfection reagent (Roche, Cambridge, MA) according to the manufacturer’s protocol for 2 days. Filtered virus particles were then used to infect HepG2 cells. All infected cells were selected in media containing 2 μg/ml puromycin. Silencing of APOB expression was evaluated by western blot analysis using anti-APOB (sc-393636; Santa Cruz Biotechnology, Minneapolis, MN) and anti-β-actin (ab3280; Abcam, Cambridge, MA) antibodies.

#### XTT cell proliferation assay

Cell proliferation was measured using a sodium 3′-[1-[(phenylamino)-carbony]-3,4-tetrazolium]-bis(4-methoxy-6-nitro)benzene-sulfonic acid hydrate (XTT) assay kit (Roche, Cambridge, MA) according to the manufacturer’s manual. Briefly, 1000–2000 cells in 100 μl of culture medium were cultured in 96-well culture dishes (Sigma, St. Louis, MO) for 72–96 h. Then, 50 μl of XTT labeling mixture was added to each well. After incubation for 6 h, the absorbance of each well was measured using an infinite M1000 Pro microreader (TECAN, Männedorf, Switzerland). The percentages of absorbance relative to those of control knockdown cells were plotted as relative cell proliferation.

### Data analysis

Collected gene expression data were transformed and normalized as described previously^21,28,32–34^. The BRB Array Tools software program (http://linus.nci.nih.gov/BRB-ArrayTools.html) was used for analysis of the gene expression data and construction of a prediction model^35^. A heat map was generated using the Cluster and TreeView software programs^36^, and further statistical analysis was performed using R language (http://www.r-project.org). When a gene was represented more than once on the microarray platform, the genes with the greatest variance in expression were selected.

Before collation of human and mouse gene expression data for clustering or construction of prediction models, expression levels of orthologous genes in the mouse and human data sets were independently standardized by transforming the expression level of each gene to a mean of 0 and a standard deviation of 1, as described in earlier studies^20,37^. To construct the prediction models, we used a Bayesian compound covariate prediction (BCCP) model as described previously to estimate the probability that a particular mouse HCC tissue would have a given gene expression signature^20,21,28,32^. Briefly, gene expression data in training sets (mouse and Samsung and Fudan cohort data) were combined to form a classifier according to BCCP. The robustness of the classifier was assessed using a misclassification rate determined using leave-one-out cross-validation in the training set. The sensitivity of predicting a subtype with low-Apob expression in the training set was 0.884, and the specificity was 0.917. The BCCP classifier estimated the likelihood that an individual mouse tumor would have either subtype of a given gene expression signature (Apob silencing or not) and dichotomized tumors according to Bayesian probability (cutoff of 0.5).

## Results

### Apob-associated gene expression data from mouse livers

**Fig. 1:**
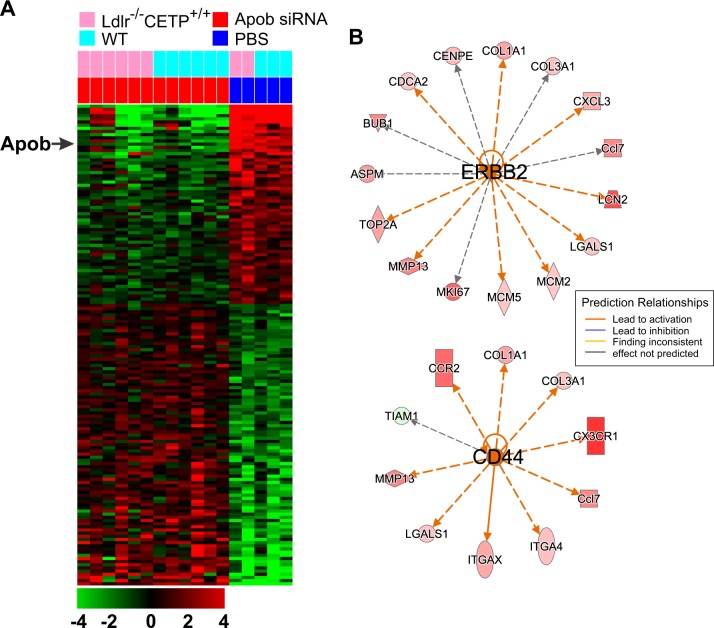
Apob-associated gene expression signature in mouse livers. **a** Gene expression data were collected after treatment of mice with two siRNAs specific for Apob or with phosphate-buffered saline (PBS) as the control. The expression of 152 genes was significantly altered (1.5-fold difference, *P* < 0.005) by silencing of Apob expression in mouse livers regardless of the genetic background and difference in siRNAs. Ldlr^−/−^ CETP^+/+^ indicates mice with the Ldlr^−/−^ mutation and the human CETP^+/+^ transgene under the human APOA1 promoter. WT indicates wild type. The data are presented in matrix format in which rows represent individual genes and columns represent tissue. Each cell in the matrix represents the expression level of a gene feature in an individual tissue. Red reflects relatively high expression and low reflects relatively low expression levels, as indicated in the scale bar (log_2_-transformed scale). Gene expression data are available in the Gene Expression Omnibus database (GSE23088). **b** Network analysis with Apob signature genes revealed that many of the genes were downstream targets of ERBB2 and CD44. Genes are color-coded according to the Apob siRNA/PBS ratio. Red represents relatively high expression in Apob-silenced liver and green represents relatively low expression in Apob-silenced liver

Because a large fraction of mutations in APOB are truncation mutations leading to underexpression of APOB (Supplementary Fig. 1), we selected gene expression data generated from mouse liver in which Apob is silenced by two different siRNAs^23^. To identify genes whose expression is associated with Apob expression in mouse liver, we applied a two-sample *t*-test to gene expression data from mouse livers treated with two Apob-specific siRNAs for Apob expression silencing and control livers from mice treated with phosphate-buffered saline. In total, 152 genes were differentially expressed between Apob-silenced and control livers (Fig. 1a). As expected, expression of Apob was significantly downregulated in livers treated with two Apob-specific siRNAs, suggesting that most of these genes are directly or indirectly associated with Apob in the liver. Many of the downregulated genes in livers with silenced Apob were related to immune activity, including *Cxcl13*, *Cd163*, *Vsig4*, *Timd4*, and *Icos*. This suggests that Apob expression might be necessary for maintaining immune cell activity in the liver. Hereafter, we refer to the gene expression signature associated with Apob silencing as the “Apob silencing signature (ASS).”

To determine the underlying biology of Apob depletion in the liver, we next carried out a gene network analysis of the Apob signature using Ingenuity Pathway Analysis. Surprisingly, network analysis revealed that many of the regulators involved in cell growth were activated in Apob-silenced livers (Table 2). Many of the upregulated genes in Apob-silenced livers are direct downstream targets of receptor tyrosine kinase Erbb2 (Fig. 1b) and growth factor Vegfa. Oncogenic transcription regulators, such as Mitf, Foxm1, and E2f1, were also activated in Apob-silenced livers (Table 2). In addition, the metastasis regulator Cd44 was activated in Apob-silenced livers (Fig. 1b). Consistent with activation of many tumor-promoting regulators, *Tp53*, a major tumor suppressor, was inactivated in Apob-silenced livers, suggesting that loss of Apob in the liver may provoke initiation of tumor development.

**Table 2:** Upstream regulators of genes in the Apob-silenced signature from mouse livers

Upstream regulator	Molecule type	State	*z*-score	Target molecules in dataset
CSF2	Cytokine	Activated	3.8	ANLN, BUB1, CCL3L3, CCR1, CD86, CDCA2, CENPE, CHAF1A, EGR2, FIGNL1, FOLR2, ICOS, ITGA4, ITGAX, KNTC1, MCM5, MKI67, MNS1, RACGAP1, Saa3, TLR1, TOP2A, TPX2
CD44	Other	Activated	2.9	Ccl7, CCR2, COL1A1, COL3A1, CX3CR1, ITGA4, ITGAX, LGALS1, MMP13, TIAM1
ERBB2	Kinase	Activated	2.9	ASPM, BUB1, Ccl7, CDCA2, CENPE, COL1A1, COL3A1, CXCL3, LCN2, LGALS1, MCM2, MCM5, MKI67, MMP13, TOP2A
PTGER2	G protein receptor	Activated	2.8	ASPM, CENPE, CEP55, KIF15, MKI67, RACGAP1, TPX2, TTK
TNFSF11	Cytokine	Activated	2.7	CCL3L3, Ccl7, CCR1, CXCL3, EGR2, RACGAP1, Saa3, TTK
RABL6	Other	Activated	2.6	BUB1, KIF23, MCM2, MCM5, TOP2A, TPX2, TTK
MITF	Transcription regulator	Activated	2.6	CEP55, CHAF1A, COL1A1, ITGA4, MCM2, MCM5, TPX2
VEGFA	Growth factor	Activated	2.5	ARG2, COL1A1, HAVCR2, ITGA4, MKI67, MMP13, TLR1
CEBPA	Transcription regulator	Activated	2.4	APOB, ARG2, CCR1, COL1A1, EGR2, GLIPR1, ITGAX, KLRC1, LCN2, LGALS1, MMP13, Orm1, PTAFR, Saa3, SCD, TIAM1
SMAD3	Transcription regulator	Activated	2.3	APOB, BMP2, CCL3L3, COL1A1, COL3A1, CXCL3, MMP13, Orm1
LEP	Growth factor	Activated	2.2	CCL3L3, COL1A1, COL3A1, Cyp2d9, EGR2, ITGAX, MMP13, Saa3, SCD
MOG	Other	Activated	2.2	CCL3L3, Ccl7, CCR1, CCR2, CD86, MKI67
TBX2	Transcription regulator	Activated	2.2	ANLN, BUB1, MCM2, MCM5, NCAPG2
TNFSF12	Cytokine	Activated	2.2	CCL3L3, Ccl7, CCR1, CXCL3, MMP13
E2F1	Transcription regulator	Activated	2.2	EGR2, KIF23, MCM2, MCM5, Pmaip1, RACGAP1, TOP2A
MAP2K1	Kinase	Activated	2.2	CCL3L3, COL1A1, COL3A1, CXCL3, EGR2, MMP13
CEBPD	Transcription regulator	Activated	2.2	CCL3L3, CXCL3, ITGAX, MMP13, PTAFR, Saa3
FOXM1	Transcription regulator	Activated	2.2	CDCA2, CENPE, MKI67, PLK4, TOP2A
HGF	Growth factor	Activated	2.1	BMP2, BUB1, COL1A1, COL3A1, KIF15, KIF20B, LCN2, MCM2, MCM5, MKI67, MMP13, PLK4, PTAFR, TPX2, TTK
ERK	Kinase	Activated	2.1	ARG2, Ccl7, CCR1, COL1A1, COL3A1, EGR2, LCN2, MMP13
MAP3K7	Kinase	Activated	2.1	CCL3L3, Ccl7, CXCL3, EGR2, SCD
TET2	Enzyme	Activated	2	Ccl7, ITGAX, MMP13, Orm1
AKT	Kinase	Activated	2	BMP2, COL3A1, EGR2, LCN2
RAF1	Kinase	Activated	2	CXCL3, MKI67, MMP13, TIAM1
KRT17	Other	Activated	2	CCL3L3, CCR1, CXCL3, MMP13
LAMA5	Other	Activated	2	CCL3L3, Ccl7, CCR1, CXCL3
PTPRJ	Phosphatase	Activated	2	CCL3L3, Ccl7, CXCL3, SLFN12L
TAL1	Transcription regulator	Activated	2	ASPM, BUB1, KIF15, MCM2
FOXO1	Transcription regulator	Activated	2	ANLN, ASPM, EFHD1, EGR2, MCM5, ME1, SCD
TNNI3	Transporter	Activated	2	CCL3L3, CCR1, CCR2, CXCL3
NUPR1	Transcription regulator	Inhibited	–2.1	ASPM, BUB1, CDCA2, COL3A1, CXCL3, KIF23, MKI67, NEIL3
TCF3	Transcription regulator	Inhibited	–2.1	ANLN, BUB1, CEP55, COL1A1, DNTT, ICOS, MKI67, PLK4, RACGAP1, Serpina3, TOP2A, TTK
AHR	Nuclear receptor	Inhibited	–2.1	1810008I18Rik, ARG2, COL1A1, COL3A1, Saa3, SCD
IL3	Cytokine	Inhibited	–2.1	BMP2, Ccl6, CCR3, CD86, EGR2, ITGAX, LCN2, MCM5, MMP13, Serpina3g
ZFP36	Transcription regulator	Inhibited	–2.1	CCL3L3, COL3A1, CXCL3, KNTC1, LCN2, TOP2A
NR1H2	Nuclear receptor	Inhibited	–2.2	Ccl7, ITGAX, PLTP, Saa3, SCD
KRAS	Enzyme	Inhibited	–2.2	COL1A1, COL3A1, EGR2, G6PD, SMPD3, TOP2A
PTX3	Other	Inhibited	–2.2	CCL3L3, Ccl7, CCR2, CX3CR1, EGR2
SCAP	Other	Inhibited	–2.2	ACSS2, CYP4F2, PMVK, SCD, STARD4
ADCYAP1	Other	Inhibited	–2.2	CCL3L3, CD86, COL3A1, CXCL3, EGR2, KIF23, MCM2
CORT	Other	Inhibited	–2.4	CCL3L3, Ccl7, CCR1, CCR2, CCR3, CXCL3
TP53	Transcription regulator	Inhibited	–2.4	ANLN, ASPM, BUB1, CCL3L3, CEP55, COL1A1, COL3A1, EGR2, FIGNL1, G6PD, GLIPR1, KIF23, KNTC1, MCM2, MCM5, ME1, MKI67, MMP13, PLTP, Pmaip1, RACGAP1, SRGAP3, STARD4, TLR1, TOP2A, TPX2, TTK

### Association of ASS with prognosis in HCC

**Fig. 2:**
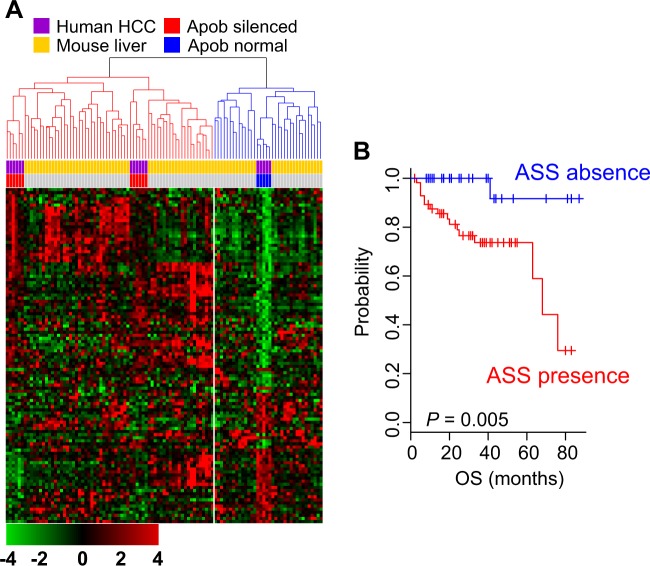
Clinical relevance of APOB ablation in hepatocellular carcinoma (HCC). **a** The Apob-silenced signature (ASS) was integrated with gene expression data from human HCC samples (The University of Texas MD Anderson Cancer Center cohort), and clustering was performed. Patients were stratified into two clusters according to their expressional similarity to mouse livers. **b** Kaplan–Meier plots of HCC patient overall survival (OS) in the MD Anderson Cancer Center cohort. Patients with the ASS had a poorer prognosis (lower OS rates) than those without the ASS

After observing that the gene expression signature reflected inactivation of Apob in the liver, we next tested the clinical relevance of the ASS in human HCC by determining the expression of ASS genes in HCC tumors and comparing the results with the mouse ASS. The clustering of pooled gene expression data revealed that HCC tumors from the MDACC cohort could be subdivided into two clusters according to their similarity to mouse livers (Fig. 2a). Of 88 HCC tumors, 57 were clustered together with the Apob-silenced mouse livers, and 31 were clustered with Apob-intact mouse livers, suggesting that APOB-silenced biological activity was mimicked in a subset of HCC tumors. In an assessment of overall survival rate in the two clusters, patients with the ASS had a significantly poorer overall survival rate than those without the ASS (*P* = 0.005 by log-rank test; Fig. 2b). This is consistent with the gene network analysis showing higher oncogenic activity in Apob-silenced mouse livers (Table 2).

**Fig. 3:**
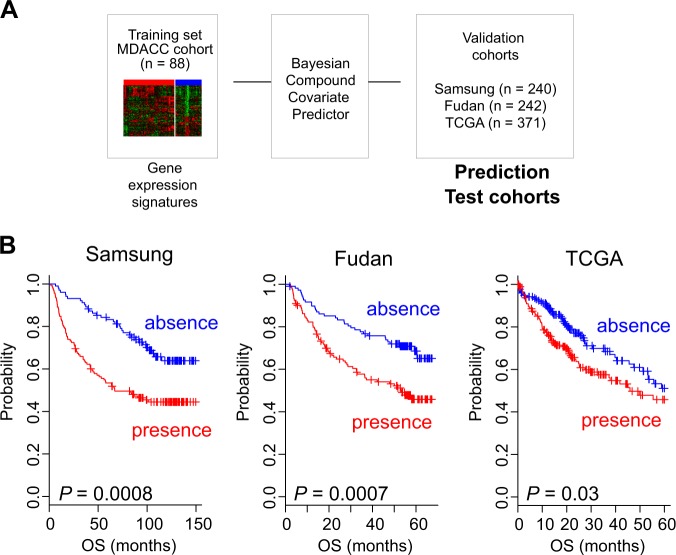
Validation of the association between the APOB-silenced signature and prognosis in hepatocellular carcinoma. **a** Schematic diagram of the prediction model. **b** Kaplan–Meier plots of patient overall survival (OS) in the validation cohorts (Samsung, Fudan, and TCGA)

To further validate the clinical association between the ASS and prognosis in HCC, we constructed a prediction model with pooled ASS data and applied the model to gene expression data from independent cohorts of patients with HCC. Briefly, the pooled ASS (training set) expression data were used to generate a BCCP that estimated the likelihood that a particular HCC tumor belonged to either subgroup (absence or presence of the ASS; Fig. 3a). When the patients were dichotomized according to the absence or presence of the ASS, tumors from about two-thirds of patients in the Samsung cohort (*n* = 240) were predicted to have the ASS, whereas tumors from the remainder were predicted to lack the ASS. Kaplan–Meier plots for the Samsung cohort showed significant differences in overall survival (*P* = 0.0008 by log-rank test) between the two subgroups (Fig. 3b), strongly indicating that APOB ablation in HCC significantly influences clinical outcome and is associated with poor prognosis.

We further tested the association of APOB ablation with clinical outcome in two additional HCC cohorts (Fudan cohort, *n* = 242; TCGA cohort, *n* = 371; Table 1). When the BCCP classifier used with the Samsung cohort was applied to these two cohorts, Kaplan–Meier plots showed significant differences in overall survival between the two predicted subgroups of both cohorts (*P* = 0.0007 for Fudan and *P* = 0.03 for TCGA cohort by log-rank test; Fig. 3b).

Taken together, these results from four independent HCC cohorts (a total of 941 patients) clearly demonstrated a strong association between APOB ablation and poor prognosis in HCC.

### Prognostic significance of APOB ablation in HCC

To evaluate the prognostic value of the gene expression signature in combination with other clinical variables, we next carried out univariate and multivariable Cox proportional hazards regression analyses with clinicopathologic variables from the Samsung and Fudan cohorts. When analyzed using American Joint Committee on Cancer stage and tumor size, which are well-known predictors of overall survival, the ASS was a statistically significant indicator of overall survival (Supplementary Table S2). In the multivariable analysis, which considered these variables together, the ASS was an independent prognostic factor for overall survival (hazard ratio = 1.459, 95% confidence interval = 1.088–1.958, *P* = 0.012).

We also assessed the independence of the prognostic signature over the current staging system. When the signature was applied to patients with different stages, it successfully identified stage I and II disease patients with poor prognosis (Supplementary Fig. S2).

Taken together, these findings suggest that the ASS retains its prognostic relevance even after the classic clinicopathologic prognostic features have been taken into account.

### Association of the ASS with previous molecular subtypes

**Fig. 4:**
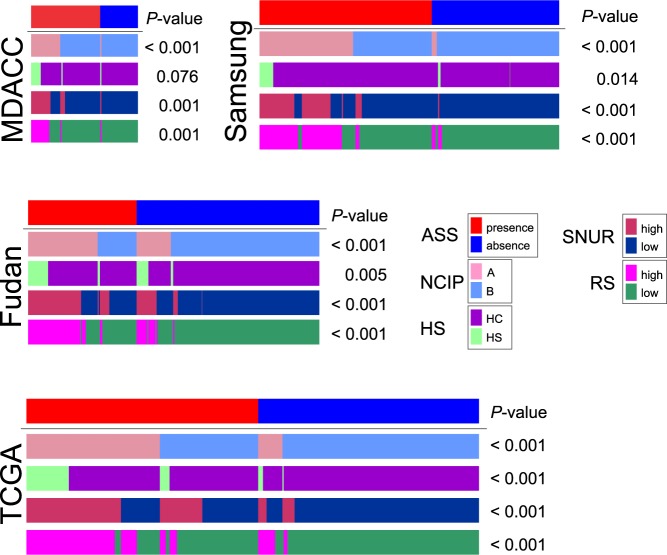
Association of the APOB-silenced signature with previously identified molecular subtypes of hepatocellular carcinoma. The significance of the associations was assessed using a *χ*^2^ test. ASS APOB-silenced signature, NCIP National Cancer Institute proliferation, HS hepatic stem cell (HS or hepatocyte [HC]), SNUR Seoul National University recurrence, RS recurrence-risk score

NCIP subtypes (A or B) were previously discovered by unsupervised analysis of gene expression data from human HCC samples, and subtype A represents tumors with a poor prognosis^20,28,29^. To assess the association between ASS and these two molecular subtypes, we stratified patients in the MDACC cohort according to the NCIP genomic signature and the grouping of patients by NCIP subtype and ASS subtype was compared. Most patients with NCIP subtype A were classified as having the ASS (*P* = 1.2 × 10^−16^, *χ*^2^ test; Fig. 4), suggesting that high-APOB activity is associated with NCIP subtype A (i.e., poor prognosis). The association between the NCIP subtype and APOB activity was also significant in the Samsung, Fudan, and TCGA cohorts (Fig. 4).

The HS gene signature (HS or hepatocyte [HC] subtypes) was previously defined as gene expression patterns resembling those found in fetal hepatic stem cells; the prognosis for patients with the HS subtype is extremely poor^21^. Not surprisingly, the presence of the ASS was associated with the HS subtype in most of the cohorts (Fig. 4).

SNUR subtypes (high-recurrence or low-recurrence) were identified using supervised approaches to select genes associated with early disease recurrence after curative-intent treatment^30^. Consistent with the NCIP results, presence of the ASS was associated with the high-recurrence SNUR subtype (*P* < 0.001 in all cohorts; Fig. 4).

RS was determined by integrating multiple prognostic signatures to identify a small number of genes that predicted recurrence after treatment^31^; scores typically range from 0 to 100 (high-recurrence subtype >50). Consistent with the results for the other subtypes, presence of the ASS was associated with the high RS subtype (*P* < 0.001 in all cohorts; Fig. 4).

Taken together, this comparison showed great concordance between the ASS and previously recognized genomic subtypes, further indicating that APOB might play an important role during HCC progression.

### Biological insights of low-APOB HCC

We next identified genes whose expression patterns were specific to the ASS and were conserved in HCC tissue samples from all four cohorts. The expression of 753 genes was significantly associated with the presence and absence of the ASS in all four cohorts. To gain a better understanding of the underlying biology of APOB ablation in HCC tumors, we applied gene network analysis from Ingenuity Pathway Analysis to this shared gene expression signature. Consistent with our analysis of the mouse ASS (Table 2), upregulated genes in APOB-ablated HCC included *HGF*, *MITF*, *MYC*, *ERBB2*, *FOXM1*, and *E2F1* (Supplementary Table S3). Also consistent with analysis of the mouse ASS, tumor suppressors, such as *TP53* and *PTEN*, were inactivated in HCC tumors with APOB ablation.

We also analyzed proteomic data from TCGA cohort to investigate proteomic characteristics of HCC according to the ASS (Supplementary Fig. S3). A regulatory subunit of PI3K (p85) and two RAF kinases (B-RAF and C-RAF) were highly expressed in HCC with APOB ablation, suggesting that these proteins might also be responsible for poor clinical outcomes in patients with APOB ablation.

### Depletion of APOB expression increases proliferation of HCC cells

**Fig. 5:**
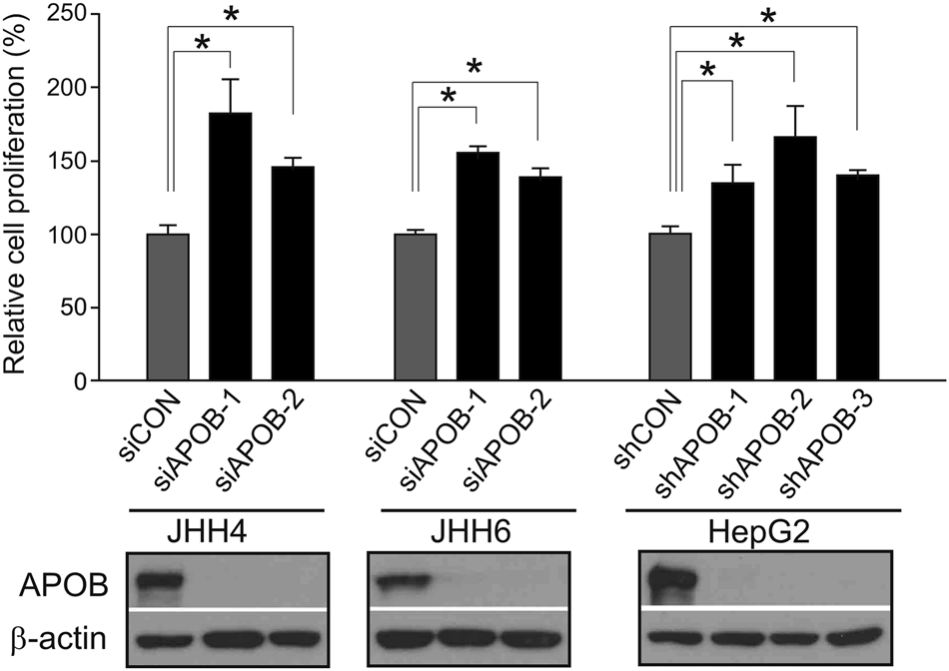
APOB ablation increases the growth of HCC cells. Depletion of APOB by siRNAs or shRNAs significantly increased HCC cell proliferation. Two siRNAs and three shRNAs were used for silencing APOB expression in JHH4, JHH6, and HepG2 cells. The expression of APOB after treatment with siRNAs and shRNAs was measured with western blotting. *P < 0.01 by Student’s *t*-test

Because APOB ablation is significantly associated with poor prognosis of HCC patients and highly correlated with expression of cell growth-stimulating genes, such as *MYC*, *FOXM1*, *HGF*, and *E2F1*, we next tested if silencing APOB expression had any effects on the proliferation of HCC cells. Interestingly, the proliferation of JHH4 HCC cells was significantly increased when expression of APOB was silenced by siRNAs (*P* < 0.01 by Student’s *t*-test, Fig. 5). Likewise, proliferation of JHH6 and HepG2 HCC cells was significantly increased upon depletion of APOB with siRNAs or shRNAs. These results are in good agreement with the clinical associations with APOB ablation in HCC and suggest that APOB may have weak potential tumor suppressive activity.

## Discussion

Although recent genomic studies identified unexpected frequent mutations in serum proteins such as ALB and APOB^17,38,39^, the clinical significance of these mutations is not known. In the present study, by applying systematic integration of genomic data from mouse models and human HCC tumors, we showed that APOB ablation in the liver is associated with poor prognosis in patients with HCC. Further analysis of multiple genomic data revealed potential links between APOB ablation and several oncogenic pathways that might account for the poor patient prognosis.

For the development of the genomic signature associated with APOB ablation in the liver and validation of its association with prognosis, we used an unsupervised approach combined with a supervised approach. Unsupervised hierarchical clustering of the mouse ASS with gene expression data from human HCC tumors identified HCC patients with APOB ablation. Then, supervised prediction models were used to validate the association between the ASS and clinical outcome in three independent patient cohorts. The high sensitivity (>0.8) and specificity (>0.9) observed during training of the prediction models within the training cohort, as well as the association between the predicted outcome and patient prognosis in three validation cohorts, indicated that the ASS was robust. In addition, the prognostic significance of the ASS was supported by the results of additional tests. The ASS could identify high-risk patients among those with early-stage tumors (stage I and II) and, in multivariable analysis, the ASS was one of the most significant predictive factors for overall survival. Taken together, these results indicate that APOB may play a critical role in the development and progression of HCC.

Although the association of APOB ablation with poor prognosis in HCC was clear in the present study, the underlying biology of this association remains unknown. The precise mechanism connecting APOB with tumorigenesis is unclear, although the biological association between obesity and cancer is already known to be related to circulating lipid levels and tissue lipid metabolism. For the first time, the present study clearly demonstrated that APOB ablation is associated with clinical outcomes in patients with HCC. Whole-exome sequencing results published recently showed substantial APOB gene mutations in HCC tumors (10%); most of these were truncating and missense mutations, with decreased mRNA expression in HCC tissues relative to normal tissues^17^. This is consistent with our findings. In addition, FHBL, which is associated with apolipoproteins and caused by mutations in *APOB* genes, results in increased risk of fatty liver, liver cirrhosis, and HCC^18,40–44^. In agreement with these observations, depletion of APOB expression significantly increased the proliferation of three HCC cell lines, further supporting the notion that APOB may play roles in initiation of hepatocarcinogenesis.

There are two isoforms of APOB, APOB-100 and APOB-48. APOB-100 is synthesized by the liver and secreted in the form of very low-density lipoprotein, which uses a large amount of cellular energy^17,45^. Because such a large amount of energy is required to form very low-density lipoprotein, it can be speculated that APOB-inactivating mutations may be selected during carcinogenesis to divert energy into cancer-related metabolic pathways.

We observed an association between PTEN loss and low-APOB activity. Our in silico analysis showed that PTEN was one of the significantly inhibited upstream regulators, and loss of PTEN function is already known to enhance insulin sensitivity and promote hepatic steatosis, steatohepatitis, fibrosis, and liver cancer^46,47^. In HCC, PTEN is mutated in around 5% and PTEN expression is downregulated in nearly half of HCC cases^48,49^. In addition, the expression of PTEN is downregulated as the cancer stage progresses^50^. Although the exact mechanism linking PTEN loss of function with APOB ablation is difficult to delineate, one study reported that the loss of PTEN activity in a knockout mouse model, as well as in the HepG2 cell line, was associated with a reduction in Apob secretion^51^. The activation of lipogenesis was increased in PTEN knockout mice, along with elevated hepatic triglyceride mass, but surprisingly, this did not lead to increased export of triglycerides in the form of Apob-containing lipoproteins. Instead, Apob secretion was significantly decreased in the PTEN knockout mice. In that study, the cause of the reduction in Apob activity was explained by constitutive activation of the PI3-kinase pathway in hepatic PTEN deficiency, leading to chronic suppression of the APOB-100 protein. Consistent with this, our proteomic data showed that PI3-kinase was activated in HCC with APOB ablation.

Analysis of proteomic data showed that activation of RAF is associated with APOB ablation. Because the therapeutic benefit of sorafenib is largely mediated through its inhibition of RAF^52^, HCC with APOB ablation might be more sensitive to sorafenib if APOB ablation leads to activation of RAF kinases. However, further research is needed to determine whether patients with APOB ablation would respond better to sorafenib than those without APOB ablation.

The etiology of liver disease that leads to HCC could have been another cause of APOB ablation in our cohorts. Most patients in our study (>85%) had hepatitis B virus infection, and Wang et al.^53^ showed that serum APOB levels were much lower in patients with chronic hepatitis B virus infection than in healthy individuals, and APOB levels were also lower in pHBV1.3-transfected HepG2 cells than in cells transfected with vector only. That study proposed that HBx might be the one of the key molecules regulating APOB synthesis, and the researchers hypothesized that HBx could increase the expression of β1,4-*N*-acetylglcusaminyl transferase (GnT-III), which induced aberrant glycosylation of APOB and disrupted APOB secretion. However, unlike other studies, in which low APOB is usually preceded by decreased microsomal triglyceride transfer protein synthesis, our study showed no reduction in MTP levels. Because lipid metabolism and cancer are tightly integrated, chronic hepatitis B virus infection may decrease APOB levels and eventually cause HCC.

The present study was based on an in silico analysis and has limitations in explaining the biology of APOB in association with HCC. APOB ablation is significantly associated with poor clinical outcome in patients with HCC and increased proliferation of HCC cells, indicating that *APOB* may have weak tumor suppressive activity. Therefore, in future in vitro and in vivo experiments, it would be worthwhile to investigate whether ablation of *APOB* in the liver contributes to tumorigenesis.

## Electronic supplementary material

Supplementary data
