# Supplementary material for: Clinical significance of APOB inactivation in hepatocellular carcinoma
Source: Exp Mol Med. 2018 Nov 14;50(11):147. doi: 10.1038/s12276-018-0174-2 (PMC6235894; doi:10.1038/s12276-018-0174-2)
Supplement: Supplementary file 1 — Supplementary data [file 12276_2018_174_MOESM1_ESM.pdf]

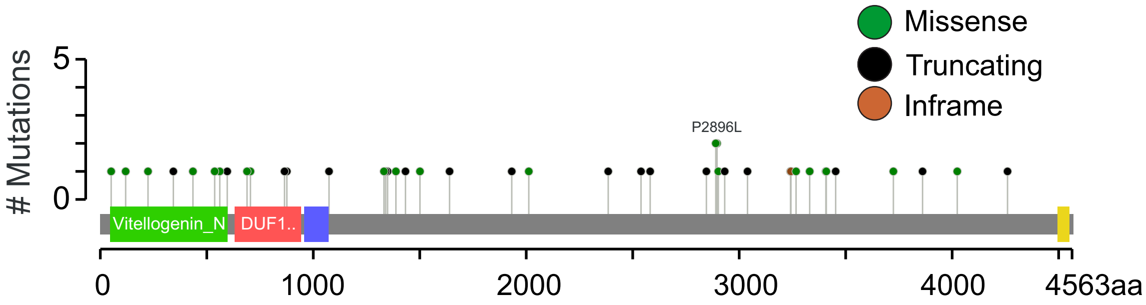

Supplementary Figure 1. Mutations of APOB in HCC from TCGA project.

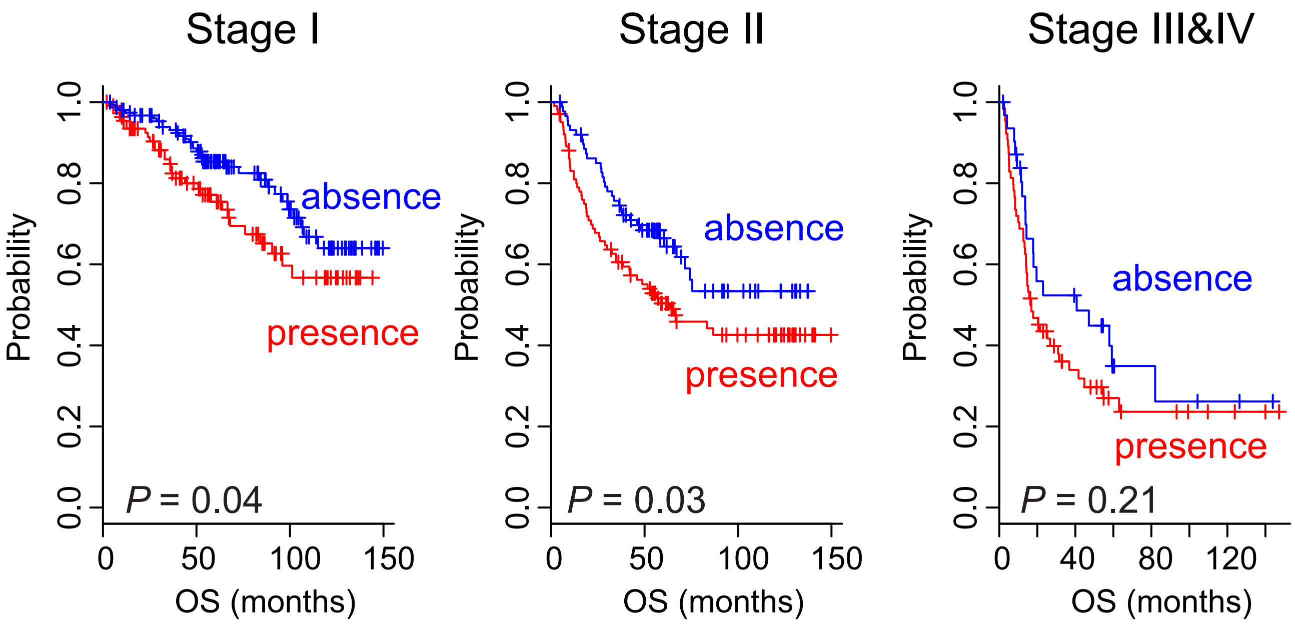

Supplementary Figure 2. Kaplan–Meier plots of overall survival (OS) in patients with hepatocellular carcinoma stratified by ASS. The prognostic significance of ASS was independent of stage. P values were obtained using the log-rank test.

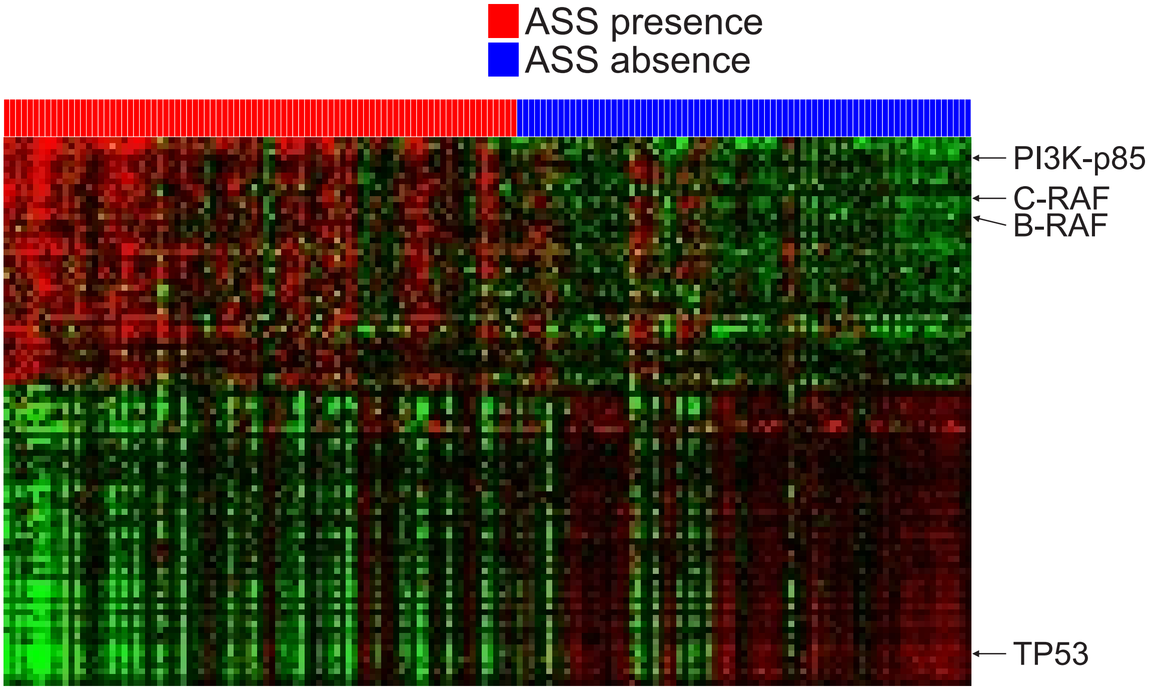

Supplementary Figure S3. Proteomic characteristics of hepatocellular carcinoma tumors associated with ASS in TCGA cohort.

Protein expression data were generated by reverse phase protein arrays. Proteins whose expression was associated with the Bayesian probability of an ASS ($r > 0.3$ or <-0.3; $P < 0.0001$) were selected (93 proteins).

Supplementary Table S1. Sequences of siRNA and shRNA used for silencing of APOB in HCC cells.

Names	Sequences
siRNAs	
Sigma mission control	
siAPOB-1	5'-GCAAGUACCUGAGAACGGATT-3'
siAPOB-2	5'-GAACAAUCCUCAGAGUUAATT-3'
shRNAs	
Sigma mission control	
shAPOB-1	5'-CCGGGAACTCAGAAGGATGGCATTTCTCGA GAAATGCCATCCTTCTGAGTTCTTTTT-3'
shAPOB-2	5'-CCGGCGGAACTATCAACTCTACAAACTCGA GTTTGTAGAGTTGATAGTTCCGTTTTT-3'
shAPOB-3	5'-CCGGCGCTACCAGAAAGACAAGAAACTCGA GTTTCTTGTCTTTCTGGTAGCGTTTTT-3'

Supplementary Table S2. Univariate and multivariable Cox proportional hazards regression analyses of overall survival in the Fudan (n = 242) and Samsung (n = 240) cohorts.

	Univariate analysis		Multivariable analysis	
**Characteristic**	**Hazard ratio (95% CI)[a]**	***P***	**Hazard ratio (95% CI)**	***P***
Age, years	0.992 (0.98-1.005)	0.225		
AFP, >300 ng/mL or ≤300 ng/mL	1.644 (1.243-2.173)	<0.001	1.041 (0.761-1.425)	0.8
Tumor size, >5 cm or ≤5 cm	1.147 (0.831-1.585)	0.405		
AJCC stage, III/IV or I/II	3.614 (2.66-4.909)	<0.001	2.284 (1.555-3.354)	<0.001
BCLC stage, B/C or 0/A	2.83 (2.134-3.753)	<0.001	1.89 (1.329-2.687)	<0.001
ASS, absence or presence	1.79 (1.32-2.3)	<0.001	1.459 (1.088-1.958)	0.012

[a]CI, confidence interval; AFP, alpha-fetoprotein; AJCC, American Joint Committee on Cancer staging system; BCLC, Barcelona Clinic Liver Cancer staging system. ASS, APOB silencing signature.

Supplementary Table S3. Upstream regulators of genes associated with ASS in hepatocellular carcinoma.

Upstream regulator	Molecule type	State	z-score
CSF2	Cytokine	Activated	6.107
RABL6	Other	Activated	5.396
HGF	Growth factor	Activated	5.31
MITF	Transcription regulator	Activated	5.131
ERBB2	Kinase	Activated	5.023
PTGER2	G protein coupled receptor	Activated	4.6
TBX2	Transcription regulator	Activated	4.571
MYC	Transcription regulator	Activated	4.491
FOXM1	Transcription regulator	Activated	4.105
ESR1	Ligand-dependent nuclear receptor	Activated	3.855
CCND1	Transcription regulator	Activated	3.68
E2F1	Transcription regulator	Activated	3.488
IFNG	Cytokine	Activated	3.442
IL1B	Cytokine	Activated	3.17
EP400	Other	Activated	3.148
AREG	Growth factor	Activated	3.144
TNF	Cytokine	Activated	3.117
CD40LG	Cytokine	Activated	2.932
MAP4K4	Kinase	Activated	2.887
SYVN1	Transporter	Activated	2.828
NR0B2	Ligand-dependent nuclear receptor	Activated	2.778
IL6	Cytokine	Activated	2.742
FGF2	Growth factor	Activated	2.724
ELAVL1	Other	Activated	2.697
ATF6	Transcription regulator	Activated	2.611
IL5	Cytokine	Activated	2.578
CCNK	Kinase	Activated	2.565
SP3	Transcription regulator	Activated	2.565
IGF1	Growth factor	Activated	2.562
CSF1	Cytokine	Activated	2.547
E2F3	Transcription regulator	Activated	2.514
CD38	Enzyme	Activated	2.448
S100A8	Other	Activated	2.433
EDN1	Cytokine	Activated	2.413
IL2	Cytokine	Activated	2.386
CNR1	G protein coupled receptor	Activated	2.335
CD24	Other	Activated	2.333
REL	Transcription regulator	Activated	2.331
EIF4E	Translation regulator	Activated	2.331
EGF	Growth factor	Activated	2.324
TAL1	Transcription regulator	Activated	2.324
SMOC2	Other	Activated	2.236

POU2F2	Transcription regulator	Activated	2.236
PCK1	Kinase	Activated	2.2
NTRK2	Kinase	Activated	2.2
S100A9	Other	Activated	2.2
EPO	Cytokine	Activated	2.186
FLT3LG	Cytokine	Activated	2.183
MKNK1	Kinase	Activated	2.121
NRG1	Growth factor	Activated	2.017
OGT	Enzyme	Activated	2
FLT1	Kinase	Activated	2
OSCAR	Other	Activated	2
ACTL6A	Other	Activated	2
YAP1	Transcription regulator	Activated	2
F3	Transmembrane receptor	Activated	2
EFNA5	Kinase	Inhibited	-2
EFNA4	Kinase	Inhibited	-2
EFNA3	Kinase	Inhibited	-2
TFRC	Transporter	Inhibited	-2
SIRT1	Transcription regulator	Inhibited	-2.008
CEBPA	Transcription regulator	Inhibited	-2.099
BNIP3L	Other	Inhibited	-2.137
AHR	Ligand-dependent nuclear receptor	Inhibited	-2.149
PTEN	Phosphatase	Inhibited	-2.156
CLDN7	Other	Inhibited	-2.219
EFNA2	Kinase	Inhibited	-2.236
EFNA1	Other	Inhibited	-2.236
SRSF2	Transcription regulator	Inhibited	-2.236
MEF2D	Transcription regulator	Inhibited	-2.236
ATP7B	Transporter	Inhibited	-2.236
RBL1	Transcription regulator	Inhibited	-2.345
NR1I3	Ligand-dependent nuclear receptor	Inhibited	-2.364
MXI1	Transcription regulator	Inhibited	-2.414
FASN	Enzyme	Inhibited	-2.433
DYRK1A	Kinase	Inhibited	-2.438
KLF15	Transcription regulator	Inhibited	-2.443
CTLA4	Transmembrane receptor	Inhibited	-2.449
MGEA5	Enzyme	Inhibited	-2.524
PPARD	Ligand-dependent nuclear receptor	Inhibited	-2.537
RXRA	Ligand-dependent nuclear receptor	Inhibited	-2.619
IRF4	Transcription regulator	Inhibited	-2.646
NLRP3	Other	Inhibited	-2.704
STAT5A	Transcription regulator	Inhibited	-2.742
GATA1	Transcription regulator	Inhibited	-2.853
CFTR	Ion channel	Inhibited	-2.867
FOXO3	Transcription regulator	Inhibited	-2.905
CDKN1A	Kinase	Inhibited	-2.917

ZFP36	Transcription regulator	Inhibited	-2.985
SPARC	Other	Inhibited	-3.051
TP53	Transcription regulator	Inhibited	-3.142
RB1	Transcription regulator	Inhibited	-3.262
PKD1	Ion channel	Inhibited	-3.302
PPARG	Ligand-dependent nuclear receptor	Inhibited	-3.402
RBL2	Other	Inhibited	-3.637
KDM5B	Transcription regulator	Inhibited	-3.651
SMARCB1	Transcription regulator	Inhibited	-3.679
HNF1A	Transcription regulator	Inhibited	-3.69
PPARGC1A	Transcription regulator	Inhibited	-3.702
NUPR1	Transcription regulator	Inhibited	-3.81
IL10RA	Transmembrane receptor	Inhibited	-3.841
CDKN2A	Transcription regulator	Inhibited	-4.077
HNF4A	Transcription regulator	Inhibited	-4.462
